# Peculiarities and management challenges of advanced renal cell carcinoma incidentally discovered in pregnancy

**DOI:** 10.1002/ccr3.1485

**Published:** 2018-03-12

**Authors:** Rotimi A. David, Boluwatife Idowu, Cathlyn Akindiose, Adeyinka Laoye, John A. Aluko, Adeleye D. Omisore, Emmanuel Alajiki, Akinwumi O. Komolafe, Abdulkadir A. Salako, Uchenna Onwudiegwu

**Affiliations:** ^1^ Urology Unit Department of Surgery Obafemi Awolowo University Teaching Hospitals Complex Ile‐Ife Nigeria; ^2^ Department of Obstetrics and Gynaecology Obafemi Awolowo University Teaching Hospitals Complex Ile‐Ife Nigeria; ^3^ General Surgery Unit Department of Surgery Obafemi Awolowo University Teaching Hospitals Complex Ile‐Ife Nigeria; ^4^ Department of Morbid Anatomy Obafemi Awolowo University Teaching Hospitals Complex Ile‐Ife Nigeria; ^5^ Department of Radiology Obafemi Awolowo University Teaching Hospitals Complex Ile‐Ife Nigeria

**Keywords:** case report, incidental diagnosis, peculiar presentation, pregnancy, renal cell carcinoma

## Abstract

Our aim is that urologists, gynecologists, nephrologists, and general practitioners will be reminded that diagnosis of renal malignancies sometimes require a high index of suspicion as they may remain asymptomatic in advanced stages; even as they can also rarely co‐exist with and cause peculiar challenges in pregnancy.

## Introduction

Renal cell carcinoma (RCC) is quite rare in pregnancy with only about fifty documented cases in world literature 1. The presence of the fetus and the absence of standardized global treatment guidelines on the subject can pose unique diagnostic and management challenges 2. Through this report, we aim to highlight the peculiarities of clinical presentation and management challenges of patients with advanced RCC incidentally diagnosed during pregnancy.

## Case Reports

We present two patients with incidentally discovered, advanced RCC co‐existing with pregnancy, after obtaining informed patient consent and ethics approval from our university teaching hospital.

**Figure 1:**
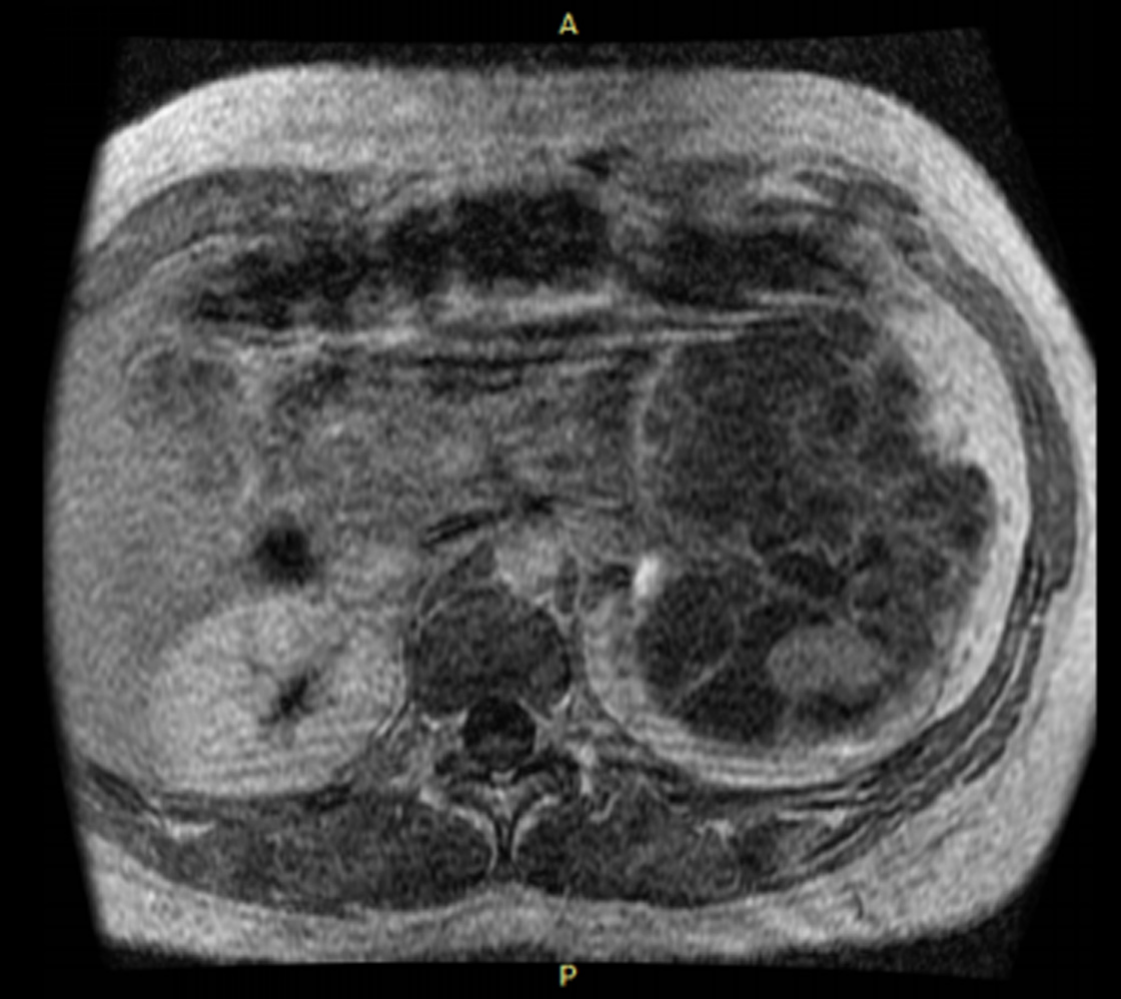
Magnetic resonance image of our first patient showing the complex cystic left renal mass.

**Figure 2:**
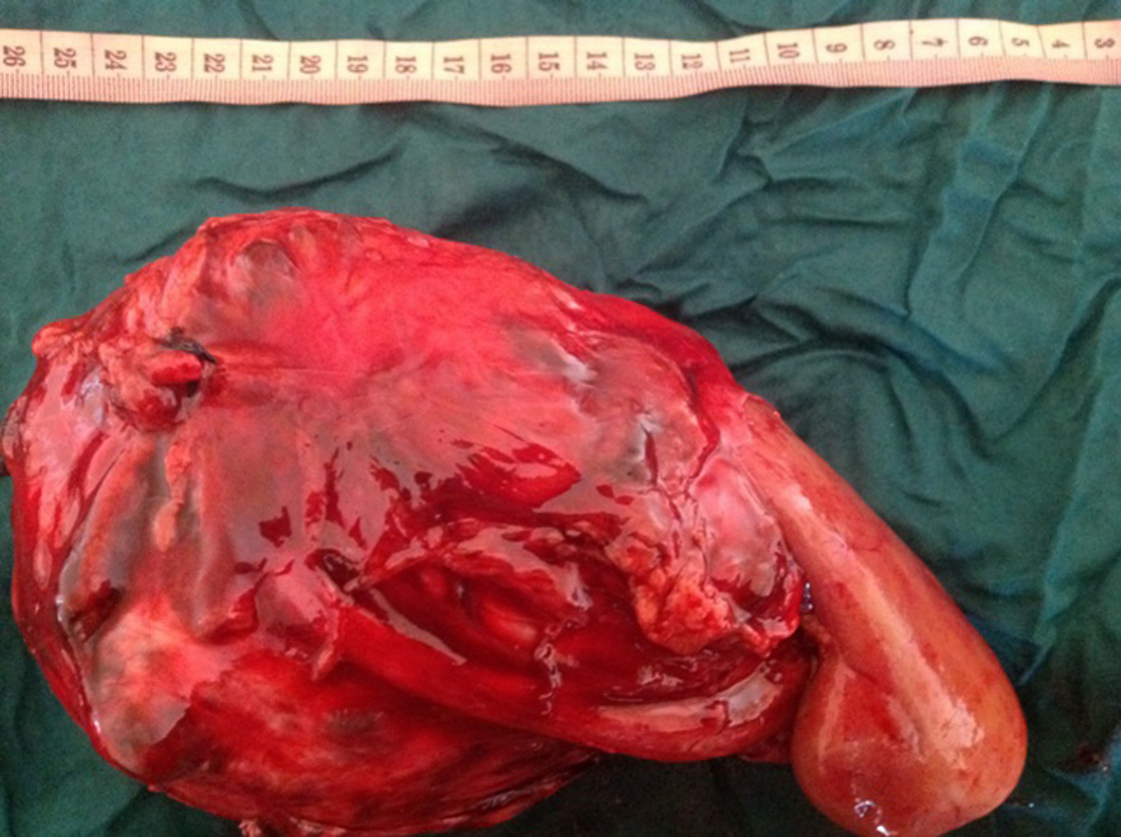
Radical nephrectomy specimen of our first patient weighing 1.1 kg.

Our first patient is a 32‐year‐old Gravida 3, Para 2 woman with incidental ultrasound finding of a complex cystic left renal mass measuring 16.1 × 5.1 cm during third trimester. Previous ultrasound in first trimester was normal. History and physical examination were unremarkable. She defaulted before further evaluation, but represented to us 5‐months after vaginal delivery in a secondary hospital. Magnetic resonance imaging (MRI) was then performed and showed Bosniak IV complex cystic left renal mass (Fig. 1) necessitating left radical nephrectomy a month thereafter (Fig. 2). Histology confirmed multilocular cystic RCC of low malignant potential (T3a, N0, M0). She was counseled on contraception and is still on routine follow‐up in our urology out‐patient clinic, 30‐months after the nephrectomy.

**Figure 3:**
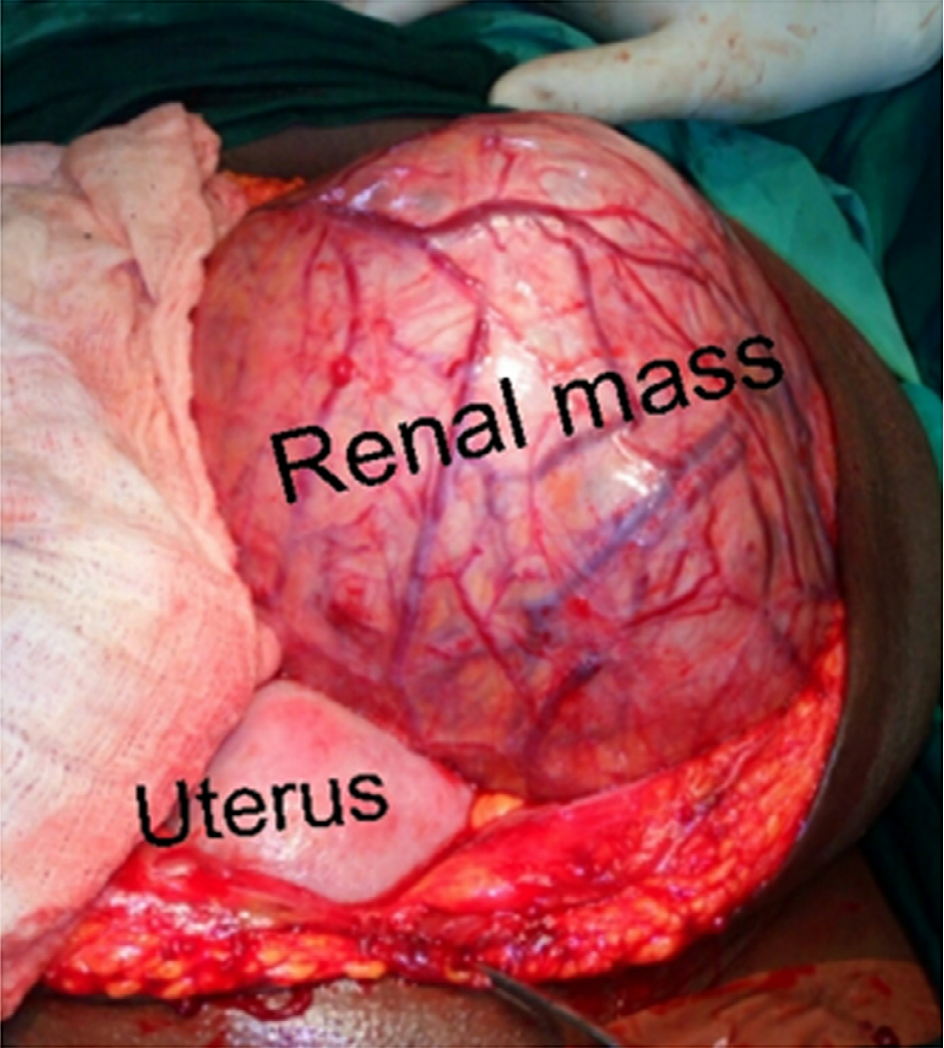
Huge, highly vascularized solid left renal mass (specimen 3.7 kg) seen intra‐operatively in our second patient.

**Figure 4:**
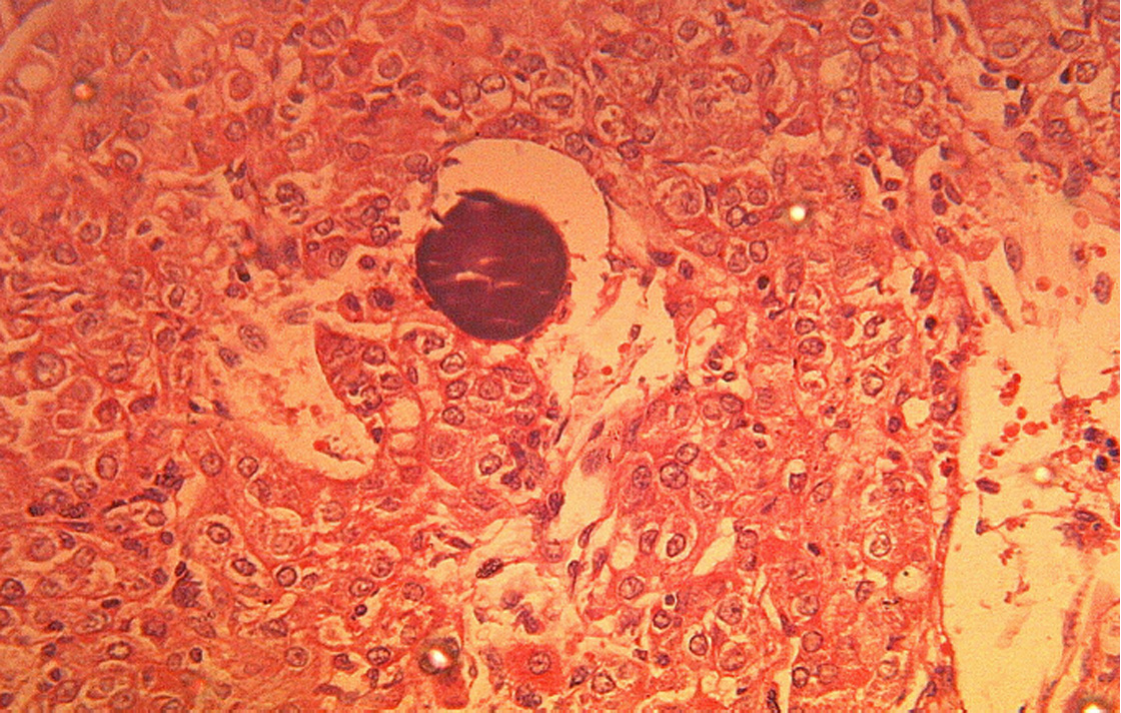
Photomicrograph of our second patient showing papillary variant renal cell carcinoma: A solid growth of highly pleomorphic tumor cells with some of the cells displaying cytoplasmic clearing. A psamomma body is present in the center (H&E ×400).

The second patient is a 35‐year‐ old Gravida 4, Para 3 woman scheduled for elective cesarean section (CS) in order to avoid a potentially difficult labor secondary to an ultrasound diagnosed, huge subserous leiomyoma co‐existing with pregnancy. History/physical examination was not contributory and renal function was normal. Intra‐operatively during the CS, a live male neonate was delivered. No leiomyoma was present. A solid mass was however seen superolateral to the uterus (Fig. 3). There were initial doubts about its organ of origin but further exploration revealed this to be a left renal mass. The right kidney was grossly normal. A decision was made to proceed with left radical nephrectomy, as there was suspected iatrogenic devascularization of the left ureter and the left renal mass during the dissection. Histology of the specimen revealed papillary RCC (T3a,N0,M0)‐ Fig. 4. She currently has no problems, has been counseled on contraception and is on regular out‐patient follow‐up, 18 months after surgery.

## Discussion

RCC is rare during pregnancy and its management when it does occur constitutes a real challenge 2. The female gender and relatively young ages of the patients presented is not overtly surprising in our domain, as we have recently documented that RCC is commoner in females of 3rd decade in our environment; contrary to experience from other parts of the world 3.

Aside from multiparity, there were no putative risk factors for RCC in both patients. The classical triad of loin pain, loin mass, and hematuria have become increasingly uncommon in RCC and is even rarer in pregnant women 3. However, as it is known that RCC may cause a myriad of features, the absence of any obvious symptom in either of our patients despite advanced/huge nature of the tumors is somewhat surprising. The physiologic changes of pregnancy and increasing abdominal girth might have masked the features; allowing the tumor escape detection till it had attained a relatively large size.

The RCC was discovered incidentally by ultrasound in our first patient while in the second patient, the scan findings were misleading, highlighting the limitations of ultrasound and need to interpret results with caution especially when the fetus has become large enough to limit visualization and distort the sonologic anatomy. There should, therefore, be a low threshold for MRI in all suspicious abdominal masses discovered by ultrasound during pregnancy. Gadolinium should however be used with caution as it can cross the placenta and induce fetal hypothyroidism 2.

The timing of diagnosis, paucity of literature and lack of standardized global treatment guidelines constituted additional management challenge. One of the women initially defaulted from our care, an unsurprising problem as it is difficult for patients in our environment to accept radical nephrectomy during pregnancy 1; with poverty, ignorance and strong beliefs in traditional medical practices further compounding this problem. Being in the third trimester, our default management approach would have been to deliver the baby by CS, resect the tumor and perform bilateral tubal ligation at same surgery. Delaying the surgery into puerperium is an alternative, as this would prevent fetal loss, reduce tumor vascularity, and improve surgical access 4. Our second patient whose tumor was discovered intra‐operatively posed significant management dilemma as we had been unable to properly stage the tumor and prepare her for radical nephrectomy before laparotomy. Our decision to go ahead and perform radical nephrectomy concurrently at the time of CS was however not out of place as it spared her from recurrent surgery/anesthesia risks; and similar action has been documented in literature 5.

Worldwide, there is no consensus on best timing for radical nephrectomy if the RCC had been discovered in first or second trimester. Termination of pregnancy is routinely not advised except in patients with rapidly growing tumors or metastatic disease 2. It is generally believed that radical nephrectomy can be safely carried out in first trimester while concerns about uterine contractions and spontaneous abortion abound if performed in second trimester 6. The relatively long doubling time of RCC (300–500‐days) 2 is reason some authors advocate for deferring the nephrectomy till delivery, although this is still an area of controversy 1, 6. Open surgery was used in our patients due to surgeon preference, large tumor size, and unavailability of advanced minimal access techniques in our environment. Open, laparoscopic, and robotic modalities have however all been reported in literature for the procedure 2, 7.

Clear cell variant was the commonest form of RCC in pregnancy from previous reports 2, 4. The RCC histologic types encountered in both of our patients are indeed rare. For unknown reasons, we are increasingly encountering uncommon RCC subtypes in our practice 8. Both patients were counseled on contraception post‐operatively due to the role of pregnancy‐related hormones in etiology of RCC 9. The oral contraceptive pills they were encouraged to use have also been associated with risk reduction of RCC 10.

The small number of patients presented is a relative limitation of our study. However, in the overall context of the rarity of RCC in pregnancy, coupled with the paucity of data on the subject, it will be appreciated that there is need to share our experiences as is being done in this report. There is, however, need for further studies in order to develop standardized global guidelines that can strongly impact routine clinical practice.

## Conclusion

Although rare, advanced RCC can incidentally co‐exist with pregnancy. Diagnosis requires a high index of suspicion and RCC should be remotely considered in the differential diagnosis of all abdominal masses in pregnancy. Ultrasound may be misleading and radical nephrectomy can be safely carried out during operative delivery or in the puerperium.

## Conflict of Interest

None.

## Authorship

RAD and BI: conceptualized and contributed to all aspects of the manuscript. CA, AL, JAA, ADO, and EA: contributed to study design and literature review. ADO: reviewed the radiologic images. JAA and AOK: worked on the histologic slides. AOK, AAS, and UO: were involved in overall supervision and finalization of the paper. All authors read and approved the final manuscript.
